# Set‐up error and dosimetric analysis of HexaPOD evo RT 6D couch combined with cone beam CT image‐guided intensity‐modulated radiotherapy for primary malignant tumor of the cervical spine

**DOI:** 10.1002/acm2.12840

**Published:** 2020-03-14

**Authors:** Ping Jiang, Xile Zhang, Shuhua Wei, Tiandi Zhao, Junjie Wang

**Affiliations:** ^1^ Department of Radiation Oncology Peking University 3rd Hospital Beijing 100191 China

**Keywords:** cervical spinal tumor, dosimetric analysis, IMRT

## Abstract

**Purpose:**

To investigate the set‐up error and consequent dosimetric change in HexaPOD evo RT 6D couch under image‐guided intensity‐modulated radiotherapy (IG‐IMRT) for primary malignant tumor of the cervical spine.

**Methods:**

Ten cases with primary malignant tumor of the cervical spine were treated with intensity‐modulated radiotherapy (IMRT) in our hospital from August 2013 to November 2014. The X‐ray volumetric images (XVI) were scanned and obtained by cone‐beam CT (CBCT). The six directions (6D) of set‐up errors of translation and rotation were obtained by planned CT image registration. HexaPOD evo RT 6D couch made online correction of the set‐up error, and then the CBCT was conducted to obtain the residual error.

**Results:**

We performed set‐up error and dosimetric analysis. First, for the set‐up error analysis, the average error in three translation directions of 6D set‐up error of the primary tumor of the cervical spine was <2 mm, whereas the single maximum error (absolute value) is 7.0 mm. Among average errors of rotation direction, Rotation X (RX) direction 0.67° ± 0.04°, Rotation Y (RY) direction 1.06° ± 0.06°, Rotation Z (RZ) direction 0.78° ± 0.05°; and the single maximum error in three rotation directions were 2.8°, 3.8°, and 2.9°, respectively. On three directions (X, Y, Z axis), the extended distance from clinical target volume (CTV) to planning target volume (PTV) was 3.45, 3.17, and 3.90 mm by calculating, respectively. Then, for the dosimetric analysis, the parameters, including plan sum PTV D98 and D95, planning gross tumor volume D98 and D95, V100% of the plan sum were significantly lower than the treatment plan. Moreover, Dmax of the spinal cord was significantly higher than the treatment plan.

**Conclusion:**

6D set‐up error correction system should be used for accurate position calibration of precise radiotherapy for patients with malignant tumor of the cervical spine.

**Abbreviations**
IMRT: Intensity‐modulated radiotherapy
RT: Radiotherapy
CT: Computed tomography
CBCT: Cone beam computed tomography
GTV: Gross tumor volume
CTV: Clinical target volume
PTV: Planning target volume
PGTV: Planning gross tumor volume

## 1 Introduction

The radiotherapy for the tumor of spine demands high techniques and equipment. Intensity‐modulated radiotherapy (IMRT), proton radiotherapy, carbon ion or other heavy ion radiotherapy equipment; stereotactic radiosurgery (SRS), or fractionated SRS (FSRT)^1^, ^2^ can maximize tumor doses while protecting normal tissues to the utmost extent. IMRT will increase the RT dose of the tumor of spine from the traditional conventional irradiation 40 ~ 50 Gy to 60 ~ 65 Gy.^3^ However, due to the physiological and anatomical characteristics of the spine which is close to the spinal cord, a strict‐dose‐limiting organ, high doses irradiation must have strict position verification, etc., to ensure the accuracy and safety of treatment. Linear accelerators equipped with image‐guided radiotherapy (IGRT) function have been applied in radiotherapy. With the continuous progress of imaging‐guided technology, the accuracy of position verification during radiotherapy is also enhancing.^4^, ^5^ There are many reports on the study of set‐up error, and different image verification techniques, different parts, different ways of position fixing and even set‐up errors in different unit are not the same. Rudat et al.^6^ used orthogonal plain film two‐dimensional verification position. After correction of translation 3D position, the radiotherapy residual error which still remained more than 5 mm in different parts including chest, abdomen, and head and neck accounted for 18%, 27%, and 10%, respectively. IMRT requires maximum error during the radiation treatment process, the set‐up error of which is usually required to be <2 mm.^7^ It has been reported that the translation 3D set‐up error of patients with vertebral metastasis was >7 mm by CBCT measurement. After error correction, the error in X (left to right), Y (head to foot), and Z (up to down) directions were 1.7 mm, 2.1 mm, and 1.3mm, 8 respectively. The successful development and application of HexaPOD evo RT 6D treatment couch and CBCT image‐guided system in clinical tumor radiotherapy is to improve the accuracy of radiotherapy by improving image‐guided technology. This paper analyzes the set‐up error by image‐guided of cervical spine patients, as well as the dosimetric changes caused by set‐up errors.

## 2 Materials and methods

### 2.1 Materials

From August 2013 to December 2014, set‐up error data and verification images of IMRT for 10 patients with primary malignant tumor of the cervical spine were collected. Among 10 patients, there were five cases of chordoma and five osteosarcoma. Four cases were without operation and six cases were postoperative residual or postoperative recurrence. The median age was 33 yrs old (aged 15–64). All patients were treated with IMRT and signed‐informed consents before treatment.

### 2.2 Positioning method

All patients were in supine position with C/B pillow, fixed by five‐fixed‐sites neck and 3 shoulder thermoplastic membrane. CT (Philips, Netherlands, Brilliance 1MBigBore CT) simulation was applied. Contrast‐enhanced CT scan was conducted and the scanning range was from the supraorbital margin to the thoracic spine 4 level. The range included the whole cervical spine and accessories (layer thickness 3 mm, 120 KV, 200 mAs). The scanned image was transmitted via the MOSAIQ network to Oncentra (Nucletron, External Beam v4. 3) treatment planning system for outlining target area and designing treatment plan.

### 2.3 Target area outlining and treatment plan development

**Fig. 1:**
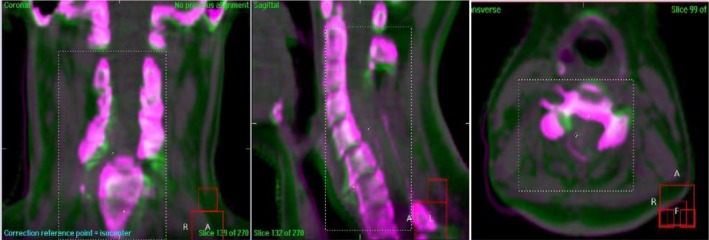
X‐ray volumetric images (pink) overlaps with planned CT images (green) (matching area in dotted box).

The target area is to be delineate the margin of the tumor on the simulated CT axial images (after the MRI image fusion of the cervical spine in the radiology department of our hospital), which is defined as gross tumor volume (GTV) (Fig. 1). Clinical target volume (CTV) refers to the lesion involving vertebral body, the accessories on both sides of the vertebral body and one vertebral body plus accessories up and down. The planning target volume (PTV) is 0.5 cm expansion of CTV. Planning gross tumor volume (PGTV) is 0.5 cm expansion of GTV. The organs at risk (OAR) are the spinal cord. After the target area outlining is confirmed, the radiotherapy plan is developed by the radiation therapy physicist. The prescription volume dose of the patients in this group is 95% PTV volume dose 44 Gy/22f, 2.0 Gy/f, and PGTV simultaneously boost to dose 60 Gy, 2.7 Gy/F.

### 2.4 Acquisition and matching of CBCT images

**Fig. 2:**
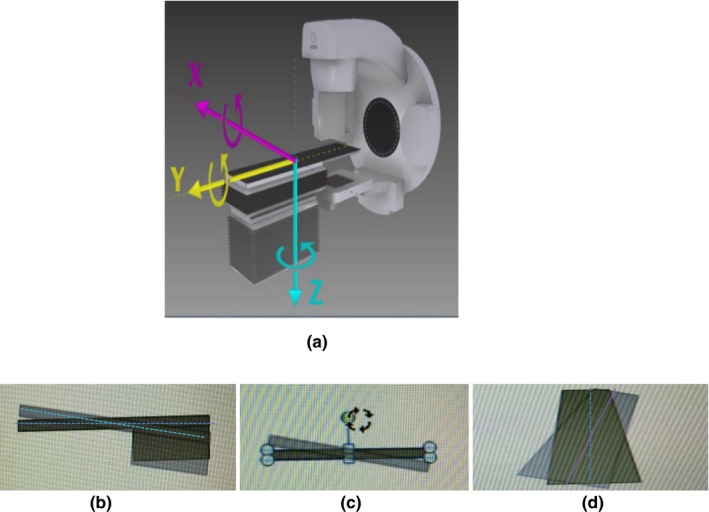
Diagram of set‐up errors in translation and rotation directions. (a) Translational direction Lateral X; Longitudinal Y; Vertical Z. (b) Rotational RX pitch. (c) Rotational RY roll. (d) Rotational RZ swing.

After applying the laser lines and marker lines of the body network in the treatment room for position set‐up, the pre CBCT scan was conducted for position verification. The scanning parameters were voltage 120 kV per frame, current 36.6 mA, collimator S20. The image of CBCT and the planning CT images were registered by automatic bone marking and manual fine tuning (Fig. 2), and confirmed by both the radiation oncologist and the radiotherapy physicist. In the Synergy coordinate system, the 6D set‐up error data could be obtained and recorded of the patient in Lateral (Lat) X, Longitudinal (Lng) Y, Vertical (Ver) Z, Rotation X (RX), Rotation Y (RY), and Rotation Z (RZ). After automatic online calibration of 6D treatment couch, again the CBCT scan was conducted again to obtain new image after correction, thus we obtain residual error in six directions after correction. During each patient’s radiotherapy course, daily CBCT verification was conducted to collect 22 pairs of CBCT images.

### 2.5 Analysis of set‐up error and external margin

The set‐up error is defined as the deviation between the image by CBCT scanning and the planned CT image in the 6D direction. Before and after correction, the CBCT scan volume images were used to analyze the inter‐fraction set‐up errors. The differences between the actual treatment position and the treatment reference position were divided into system error and random error. According to the method defined by Van Herk, the system error is the mean of the error of each patient throughout the treatment process, the sample amount of which is equal to the number of cases, with ∑ representing the standard deviation (SD) of the system error. The random error is the total error per patient minus the system error of the patient, with the sample volume equal to the product of the number of cases and the number of validations, and σ representing the standard deviation of the standard error is sigma [44].The calculation formula for the margin of planning target volume (MPTV) is defined as follows: MPTV = 2Σ total +0.70σ total, and the external margin values of CTV in the X, Y, and Z axes were calculated separately when the PTV was outlined.

### 2.6 Position error data acquisition and dosage calculation

**Fig. 3:**
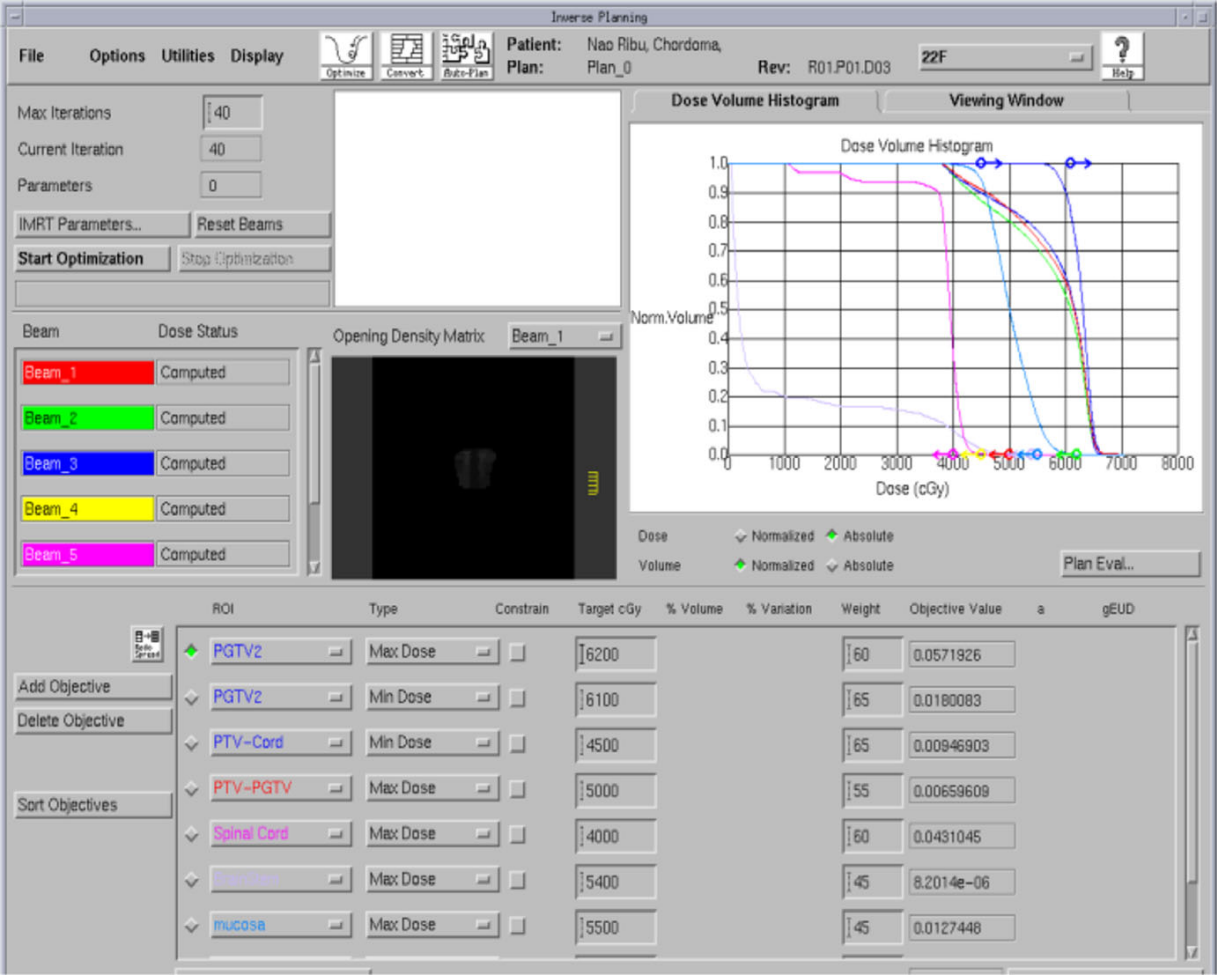
Curve‐sectional view of design dose in the treatment plan for Pinnacle treatment plan system.

**Fig. 4:**
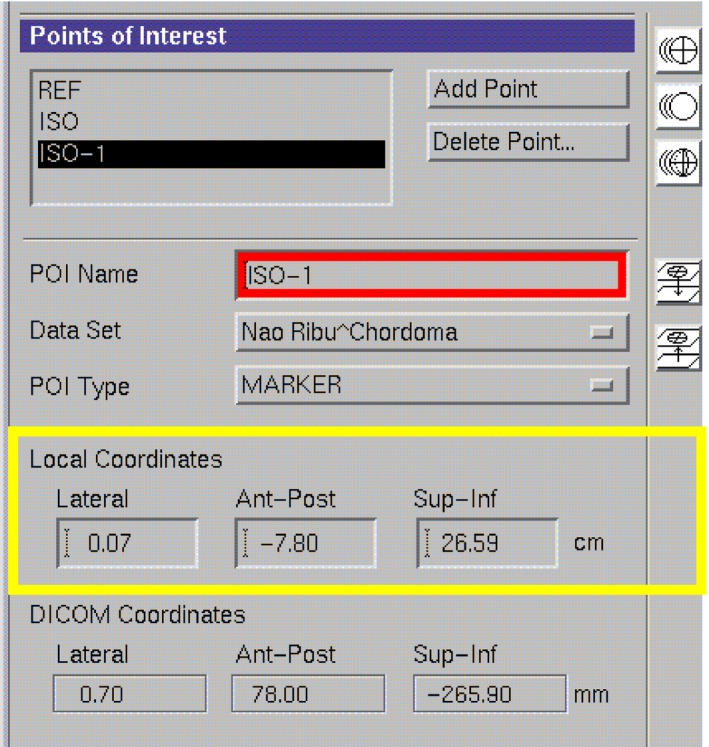
Simulate translation error of changing radiation field and other central parameters (in yellow box).

**Fig. 5:**
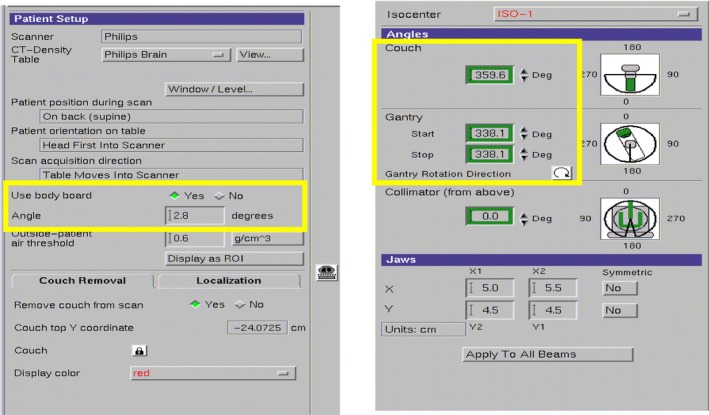
To simulate rotation error by changing couch plate angle, rack angle, and rotation treatment couch parameters (in yellow box).

**Fig. 6:**
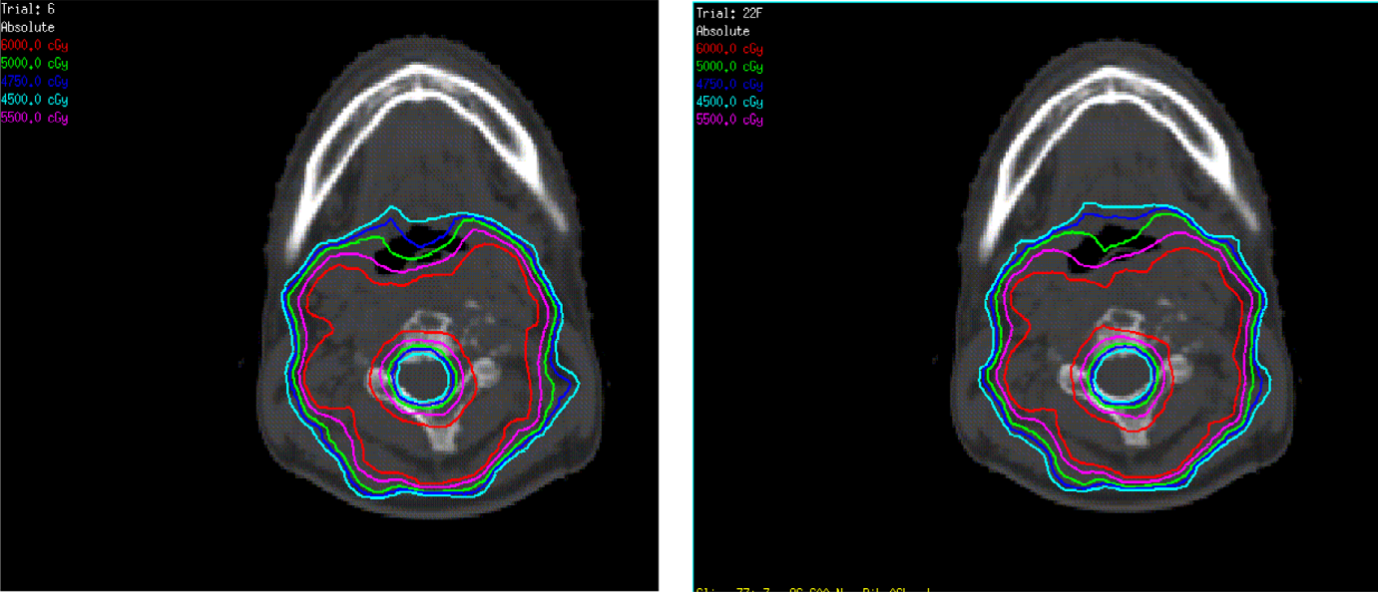
Isodose distribution map of the treatment plan (left) and the plan sum (right) Red (60Gy); purple (55Gy); green (50Gy); dark blue (47.5Gy); light blue (45Gy).

Ten cases of primary tumor of the cervical spine received CBCT scan to obtain position verification images during radiotherapy before each treatment. In the Synergy coordinate system, the 6D set‐up error data could be obtained and recorded of the patient in translational X, Y, and Z, rotational RX, RY, and RZ (Fig. 3).The patient completed the treatment according to the treatment process after correcting the errors with HexaPOD evo RT 6D treatment couch. The planning CT scan images were transmitted to the Pinnacle treatment plan system of the hospital (Fig. 4) for target area outlining and treatment planning design. The planning design was using static 7 field IMRT technology, with the patient prescription dose of 95% PTV volume dose 44 Gy/22f, 2.0 Gy/f, PGTV simultaneously boost dose of 60 Gy/22f, 2.7 Gy/f, and spinal cord dose of Dmax <45 Gy. This plan was defined as the original treatment plan in this research. According to the set‐up error of patients in each treatment, the dose of set‐up error in six directions was recalculated. Among this, the translation error was realized by changing the central parameters such as radiation field (Fig. 5), whereas the rotation error was simulated and achieved by changing the couch plate angle, rack angle, and rotation treatment couch (Fig. 6). A new total treatment plan was obtained by superimposing planning dose of which has been calculated for 22 times, that was defined as a set‐up error plan sum in this study.

### 2.7 Dosimetry observation indicators

Planning target volume (PTV) 95% volume dose (D95), 98% PTV volume dose (D98), planning gross tumor volume (PGTV) 95% volume dose (D95), and 98% PGTV volume dose (D98). Maximum dose of spinal cord (Dmax), 1cc volume dose of spinal cord (D1cc), volume dose of spinal cord (D2cc), maximum dose of oral mucosa (Dmax), and average dose of oral mucosa (Dmean).

### 2.8 Statistical processing

The data were expressed in x ± s. SPSS 16.0 software was used to analyze the data of set‐up errors and residual errors; and the set‐up error plan sum and treatment plan dosimetry parameters. If the analyzed groups of data conformed to the normal distribution, then the paired T test was adopted; otherwise, Wilcoxon rank‐sum test was used.

## 3 Results

### 3.1 The set‐up error of CBCT scanning images after matching with CT positioning images

With two times of CBCT scans, before and after correction, a total of 169 times were conducted and 338 CBCT images were collected. After testing, the error data obtained before correction were defined as set‐up error and those after correction were residual error, both of which have normal distribution. The average error of the 6D set‐up error of the primary tumor of the cervical spine (Table 1) was <2 mm in three translation directions, with single maximum error (absolute value) of 7.0 mm. Average error in rotation direction: RX 0.67° ± 0.04°, RY 1.06° ± 0.06°, and RZ 0.78° ± 0.05°. The single maximum error of three rotation directions was 2.8°, 3.8°, and 2.9°, respectively. The images obtained by the two times of CBCT scans before and after set‐up were matched with the set‐up error data and residual error data obtained by the planning reference image, matching to run repaired T test, respectively. The difference in the 6D test results was statistically significant (Table 2).

**Table 1:** Set‐up error analysis of 169 times of CBCT scan for 10 cases.

Direction	Set‐up error	Residual error	Max	Min
	$\overset{-}{x}$ ±s	$\overset{-}{x}$ ±s		
X (mm)	1.71 ± 0.10	‐0.19 ± 0.50	6.4	0.1
Y (mm)	1.81 ± 0.11	0.32 ± 0.50	7.0	0
Z (mm)	1.94 ± 0.09	‐0.44 ± 0.50	5.5	0.2
RX (°)	0.67 ± 0.04	‐0.05 ± 0.44	2.8	0
RY (°)	1.06 ± 0.06	0.18 ± 0.33	3.8	0
RZ (°)	0.78 ± 0.05	0.02 ± 0.29	2.9	0

**Table 2:** Cone‐beam CT matching result T test before and after 169 times of set‐up errors correction for 10 cases.

Direction	Translation direction			Rotation direction		
	X	Y	Z	RX	RY	RZ
T	‐5.785	4.717	2.876	‐2.27	4.109	2.057
P	0.00	0.00	0.01	0.03	0.00	0.04

*P* < 0.05 refers to the difference is statistically significant.

### 3.2 Calculation result of CTV to PTV external expansion margin

The results are shown in Table 3. According to the set‐up error, after formula calculation, the external expansion margin MPTV was 3.45, 3.17, and 3.90 mm in the direction of X, Y, and Z axes, respectively. If no error correction was done after the set‐up, the external expansion margin of CTV to PTV of the cervical spine was 4 mm in every direction. The MPTV was 1.81, 2.43, and 1.37 mm in the direction of X, Y, and Z axes, respectively, according to the residual error (after 6D couch correction). If the image‐guided CBCT was used to verify the position and correct errors, the external expansion margin of CTV to PTV can be reduced to 1.64 mm in the X direction, 0.74 mm in the Y direction, and 2.53 mm in the Z direction, on the basis of the difference in the set‐up error MPTV and the residual error MPTV.

**Table 3:** Calculation of Gross MPTV before and after 6D couch correction (mm).

Direction	Set‐up error			Residual error			Difference of the set‐up error MPTV and the residual error MPTV
	Σ_sum_	Σ_sum_	MPTV	Σ_sum_	Σ_sum_	MPTV	
X	1.33	1.12	3.45	1.12	0.98	1.81	1.64
Y	1.24	0.99	3.17	0.86	1.02	2.43	0.74
Z	1.56	1.12	3.90	0.76	0.87	1.37	2.53

MPTV, margin of planning target volume.

MPTV = 2Σsum + 0.7σsum.

### 3.3 Impact of set‐up error on the dose–volume histogram parameters of PTV and some organs at risk

**Fig. 7:**
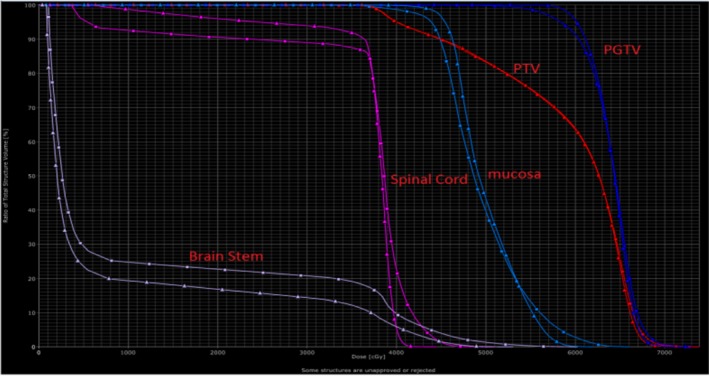
Dose–volume histograms map of tumor target area (planning gross tumor volume and planning target volume) and organs at risk (spinal cord and brainstem) for one patient in the treatment plan (▲) and plan sum (■).

Ten patients with primary tumor of the cervical spine, the prescription dose 95%PTV volume dose 44 Gy/22f, 2.0 Gy/f, PGTV simultaneously boost dose to 60 Gy/22f, 2.7 Gy/f. According to the 22 times of set‐up error data, the simulation of 22 times of treatment plan was superimposed again to generate the set‐up error plan sum. There was a difference between the plan sum and treatment plan dose distribution (Fig. 7). In the plan sum, the D98 and D95 of the dosimetry parameter PTV, as well as the D98 and D95 of PGTV was significantly lower than the original treatment plan (Tables 4 and 5).There were differences in patient’s plan sum the treatment plan dose–volume histograms (DVH) (Fig. 7).The set‐up error will result in an average reduction in 2.71 and 2.98 Gy of PTV D98 and D95, respectively, and a missing volume of average PTV 8.46%. In particular, the V100% of plan sum PGTV was significantly lower than that in the treatment plan, leading to a missing volume of average PGTV 17.48%, with the difference in statistical significance (F = 6.764, p = 0.018).

**Table 4:** Dose–volume histogram parameters of PTV in plan sum and treatment plan of set‐up error for 10 patients with primary tumor of the cervical spine.

N	PTV		PTV		PTV		PGTV		PGTV		PGTV	
	Plan sum	Treatment plan	Plan sum	Treatment plan	Plan sum	Treatment plan	Plan sum	Treatment plan	Plan sum	Treatment plan	Plan sum	Treatment plan
	D95 (Gy)	D95 (Gy)	D98 (Gy)	D98 (Gy)	V100 (%)	V100 (%)	D95 (Gy)	D95 (Gy)	D98 (Gy)	D98 (Gy)	V100 (%)	V100 (%)
1	34.87	42.80	39.24	41.05	45.76	61.48	56.34	59.87	53.86	57.46	14.12	90.97
2	44.90	45.31	42.81	43.54	56.91	57.55	59.33	59.70	56.92	57.84	93.34	94.19
3	40.84	40.37	39.52	39.82	27.52	29.10	60.20	60.72	56.97	59.99	96.13	97.96
4	46.72	48.05	44.57	45.48	9.60	37.18	53.53	56.20	50.75	53.58	17.40	66.20
5	45.69	48.61	43.05	47.70	77.16	87.80	56.67	58.32	49.78	56.89	87.64	96.88
6	43.02	43.50	39.53	41.02	57.01	62.08	52.38	54.50	47.86	51.20	83.68	89.72
7	37.10	40.72	36.26	39.89	23.32	33.70	48.33	54.38	47.24	53.03	80.87	90.91
8	35.80	42.59	34.66	41.47	41.34	42.95	50.99	56.71	51.67	54.16	68.45	84.39
9	40.01	42.60	59.33	62.78	97.42	98.47	60.92	64.33	60.63	63.04	100	100
10	40.67	43.95	39.89	43.20	47.89	58.19	54.32	57.34	51.34	55.12	79.67	84.85

PTV, planning target volume; PGTV, planning gross tumor volume.

**Table 5:** Dose–volume histogram parameters comparison of PTV in plan sum and treatment plan of set‐up error for 10 patients with primary tumor of the cervical spine.

Plan	PTV			PGTV		
	D95(Gy)	D98(Gy)	V100%	D95(Gy)	D98(Gy)	V100%
Original plan	43.85 ± 2.76	44.60 ± 6.87	56.85 ± 22.67	55.30 ± 4.14	56.23 ± 3.55	89.61 ± 9.74
Re‐plan	40.96 ± 4.15	41.89 ± 6.83	48.39 ± 25.83	58.21 ± 3.06	52.70 ± 4.33	72.13 ± 31.06
Difference	2.98 ± 2.62	2.71 ± 2.04	8.46 ± 8.51	2.91 ± 1.91	3.53 ± 1.76	17.48 ± 25.26
F	1.809	0.000	0.094	1.368	0.559	6.764
P	0.195	0.997	0.762	0.257	0.464	0.018

PTV, planning target volume; PGTV, planning gross tumor volume.

There was no statistically significant difference in dose differences between D1cc and D2cc in the spinal cord generated by errors (Tables 6 and 7).The set‐up error resulted in an increase in the irradiated dose of the spinal cord D1cc and D2cc, an average increase in 1.85 and 2.68 Gy. The plan sum spinal cord Dmax was higher than the treatment plan, with an average increase in 3.18 Gy doses, and statistically significant difference (F = 1.915, *P* = 0.046). In plan designing, the spinal cord intake dose was an organ with strict limit in dose, with prescription dose limited to Dmax <45 Gy. The set‐up error increased the risk of spinal cord radiation injury and reduced the approval rate of the plan.

**Table 6:** Dose–volume histogram parameters of organs at risk in plan sum and treatment plan of set‐up error for 10 patients with primary tumor of the cervical spine.

N	Spinal cord						Mucosa		Mucosa	
	Plan sum	Treatment plan	Plan sum	Treatment plan	Plan sum	Treatment plan	Plan sum	Treatment plan	Plan sum	Treatment plan
	D_1cc_	D_1cc_	D_2cc_	D_2cc_	D_max_	D_max_	D_mean_	D_mean_	D_max_	D_max_
1	42.88	44.36	42.34	44.09	44.54	45.73	41.92	44.62	60.12	63.59
2	44.05	42.75	43.25	42.13	45.58	44.28	52.47	52.22	64.25	64.61
3	42.62	41.64	42.26	41.27	48.50	43.25	25.59	24.77	50.22	48.75
4	41.54	41.24	40.78	40.53	47.61	42.45	33.31	33.02	55.62	59.26
5	43.25	41.67	42.07	40.82	45.59	43.14	27.94	30.35	49.59	52.25
6	44.23	43.94	43.68	43.54	46.96	45.81	37.51	33.84	62.17	63.59
7	38.80	41.58	38.08	41.13	44.01	42.66	35.78	33.67	56.78	55.89
8	46.89	43.86	45.80	43.46	48.76	43.55	42.06	40.25	60.89	61.26
9	30.84	25.17	27.01	42.79	46.01	31.10	26.31	25.77	64.75	63.01
10	45.55	44.49	44.28	44.15	45.88	45.37	43.09	45.54	65.78	63.66

**Table 7:** Dose–volume histogram parameter comparison of organs at risk in plan sum and treatment plan for the set‐up error in 10 patients with primary tumor of cervical. spine ( $\bar{x}$ ±s).

Plan	Spinal cord			Oral mucosa	
	D1cc	D2cc	Dmax	Dmean	Dmax
Treatment plan	41.07 ± 5.73	38.51 ± 12.21	42.73 ± 4.27	36.41 ± 8.98	59.59 ± 5.51
Plan sum	42.07 ± 4.51	40.96 ± 5.32	47.54 ± 1.82	36.60 ± 8.61	59.02 ± 5.81
difference	1.85 ± 1.62	2.68 ± 4.70	3.18 ± 4.37	1.71 ± 1.17	1.81 ± 1.17
F	0.010	1.101	1.915	0.082	0.065
P	0.922	0.308	0.046	0.778	0.801

Due to the set‐up error, the tendency of the dosage data of oral mucosa was not obvious. The deviation of the position was uncertain to the irradiated dose increase or reduction in oral mucosa and there was no statistical difference between the dosimetry parameters Dmean and Dmax.

## 4 Discussion

With the development of the accelerator, International Commission on Radiation Units and Measurement (ICRU) has defined two “margins” for radiation therapy programs to accurately irradiate diseased sites: the Internal Margin (IM) mainly for the movement and deformation of the organ, and the Set‐up Margin (SM) mainly for the change in the target area caused by the daily set‐up error of the patient. The concept of PTV generally includes CTV, IM, and SM. Precise position verification and effective error correction can reduce SM and unnecessary irradiation range, which is critical for situations where OAR are close to PTV.^6^ In this study, the fixed device for the radiotherapy of primary tumor of the cervical spine was the same as that for head and neck tumors. The ideal prescription dose steep reduction from vertebral tumor (GTV) to the margin of spinal cord (OAR) can be obtained by modern IMRT. Guo Leiming et al.^9^ reported the set‐up errors during EBRT for cervical spine with maximum values in RX, RY, and RZ directions were 4.9°, 7.0°, and 6.0°, and rotation errors were <2° after correction by HexaPOD evo RT 6D bed. While only 10 cases (2 cases of cervical spine, 4 thoracic spine and 4 lumbar spine) were included in the study. This study also analyzed the intra‐fraction errors during radiotherapy and the results showed that the position of patients remained stable and the intra‐fraction displacement was small in the course of treatment, which was consistent with other studies.^3^ Therefore, the intra‐fraction error of radiotherapy for vertebral tumor was not analyzed in our study. Gilbeau et al.^10^ reported that fixed thermoplastic membrane be used in radiation immobilization of the lower neck or upper clavicle area, the translation errors were between 2 to 5 mm. Data showed that this kind of fixed device can remarkably reduce the set‐up error.^11^ In our study, patients with tumors of the cervical spine used this kind of fixed device (5‐point fixed) and three translation direction set‐up error obtained produced average error <2 mm, which is consistent with the results in literatures. If the rotation error is not taken into account, it can be considered that the tumor of the cervical spine was best in set‐up repeatability, compared to the thoracic spine and lumbar spine. While Guckenberger et al.^12^ found that diminished translation errors did not lead to a reduction in rotation error. In our study, the single biggest rotation error of the tumor of the cervical spine in three rotation directions (pitch RX, roll RY, and swing RZ) were 2.8°,3.8°, and 2.9°, respectively, in which the average error of RY direction was 1.06 ± 0.06°. Set‐up error data and the residual error data were conducted with paired T test. The test results in six directions were statistically significant. As a result, we believed that the rotation error cannot be ignored in radiotherapy of tumor of the cervical spine, and 6D set‐up errors should be corrected. At present, HexaPOD evo RT 6D treatment couch can meet the clinical needs and make effective error correction. Most studies presented that head and neck tumors have accuracy of up to 0.3 mm and 0.3°.^13^

At present, IGRT combined with 6D couch on the PTV target area external expansion margin (SM) of tumor of the cervical spine has not been reported. As there is no external formula to study the rotation error, this study only considered the translation error. By formula calculation, external expansion margin MPTV in X, Y, and Z axis directions were 3.45, 3.17, and 3.90 mm, respectively. If there is no error correction after set‐up, the external expansion margins of cervical spine external irradiation CTV to PTV were 4 mm in all directions. Based on the residual error (after 6D couch correction), the MPTV was 1.81, 2.43, and 1.37 mm in the X, Y, and Z axes, respectively. If the image‐guided CBCT is used to verify the position and run error correction, the CTV to PTV external expansion margin can be reduced to 1.64 mm in the X direction, 0.74 mm in the Y direction, and 2.53 mm in the Z direction, according to the difference between the set‐up error MPTV and the residual error MPTV.

Reports on dosimetry changes brought about by radiotherapy set‐up errors are concerned with head and neck tumors such as nasopharyngeal tumors,^14^, ^15^, ^16^, ^17^ intracranial tumors,^18^ abdominal pelvic tumors,^19^ etc. The dosimetry changes generated by vertebral set‐up error are only related to the study on re‐irradiation.^20^ The side effect of radiotherapy is directly related to the dose of irradiation, so radiotherapy for tumor of the spine is often limited to low‐dose palliative treatment, which makes it difficult to obtain satisfactory therapeutic effect. The key to improve the control rate of the disease is protecting important vertebral organs such as the spinal cord, while increasing the dose of local irradiation. IMRT can increase the radiotherapy dose for the spine, whereas the dose gradient changes steeply. Consequently, it has a very high demand for treatment accuracy. Traditional radiotherapy relies solely on the body surface marker line as a reference for the treatment set‐up**.** Therefore, large errors occur and generally exist between 0.5 and 1 cm, which is unacceptable for precise treatment. Set‐up error of 1 cm may cause the spinal cord to fall into the high dose area, resulting in serious consequences [81]. Gröger et al.^20^ studied 10 cases of thoracic metastatic tumor (irradiation range 1–5 vertebral body) and discovered that average set‐up error was 6.1 ± 4 mm in translation direction and 2.7° ± 1.1° in rotation direction. Comparison of dosimetry parameters without set‐up error correction and after set‐up error correction in simulation IMRT: CTV95% prescription dose target areas were 81.6% and 86.6% respectively; PTV95% prescription dose target area were 79.4% and 89.9%; average maximum dose point of the spinal cord D0.1cc were 20.4 and 18.0 Gy. It was concluded that the set‐up error of patients in thoracic tumor radiotherapy could significantly affect the accuracy of volume dose in the target area and increase the high dose of the spinal cord. Relying only on the skin marking mode for regular set‐up to conduct radiotherapy will significantly increase the risk of radioactive myelopathy in patients. Because the set‐up error of the cervical spine tumor is smaller than that of the thoracic spine tumor, there is no local report or abroad on the dosimetry study of set‐up error in radiotherapy for primary tumor of the cervical spine. Our study recorded the translation and rotation 6D set‐up errors of primary tumor of the cervical spine in the actual fraction treatment process. Offline, by changing the irradiation field and other central parameters, the angle of the couch plate, rack angle, and rotation treatment couch, the dose distribution without errors correction was simulated and achieved. Moreover, the fraction plan was re‐superimposed to get plan sum. Compared with the treatment plan, it is found that the PTV D98 and D95 of large field radiotherapy containing CTV were reduced by an average of 2.71 and 2.98 Gy, resulting in a missing volume of average PTV 8.46%. In particular, the V100% of plan sum PGTV was significantly lower than that in the treatment plan, leading to a missing volume of average PGTV 17.48%. PGTV is the lesion area of IMRT synchronized dose, as well as the key of tumor local control. Same in the situation where set‐up error exists, the irradiated dose of spinal cord D1cc and D2cc increased, with an average increase in 1.85 and 2.68 Gy. The spinal cord Dmax was higher than that in the treatment plan, with an average increase in 3.18 Gy dose, which increased the risk of severe radioactive myelitis. In the radiotherapy of the cervical spine, oral mucosa should be properly protected. This study found that the set‐up error on the Dmax and Dmean of oral mucosa was uncertain: dose in some patients increased and some reduced. Considering the tumor’s distance to the mucosa is the key factor, the impact of the set‐up error on the mucosa volume dose difference requires further study.

## 5 Conclusion

Translation and rotation set‐up errors of cervical spine IMRT should be corrected, HexaPOD evo RT 6D can effectively correct the 6D errors. The set‐up errors can lead to insufficient dose of target volume, and increase the spinal cord dose of OAR.

## Conflict of interest

The authors declare that they have no conflict of interest.

## Funding

This study was supported by National Natural Science Foundation of China (No.61631001).

## Authors' contributions

Junjie Wang designed this study, reviewed this manuscript, and provided funding support. Ping Jiang collected and analyzed the data and drafted this manuscript. Xile Zhang designed the treatment plan of radiotherapy and assisted data analysis. Shuhua Wei collected data and reviewed this manuscript. Tiandi Zhao assisted the process of treatment.

## Data Availability

The authors declare that all data supporting the findings of this study are available within the article.
